# Inferring the Perturbed microRNA Regulatory Networks in Cancer Using Hierarchical Gene Co-Expression Signatures

**DOI:** 10.1371/journal.pone.0081032

**Published:** 2013-11-20

**Authors:** Jin Gu, Zhenyu Xuan

**Affiliations:** 1 MOE Key Laboratory of Bioinformatics; Bioinformatics Division/Center for Synthetic and Systems Biology, Tsinghua National Laboratory for Information Science and Technology, Department of Automation, Tsinghua University, Beijing, China; 2 Department of Molecular and Cell Biology, Center for Systems Biology, University of Texas at Dallas, Richardson, Texas, United States of America; Centro de Investigacion Principe Felipe, Spain

## Abstract

MicroRNAs (miRNAs), a class of endogenous small regulatory RNAs, play important roles in many biological and physiological processes. The perturbations of some miRNAs, which are usually called as onco-microRNAs (onco-miRs), are significantly associated with multiple stages of cancer. Although hundreds of miRNAs have been discovered, the perturbed miRNA regulatory networks and their functions are still poorly understood in cancer. Analyzing the expression patterns of miRNA target genes is a very useful strategy to infer the perturbed miRNA networks. However, due to the complexity of cancer transcriptome, current methods often encounter low sensitivity and report few onco-miR candidates. Here, we developed a new method, named miRHiC (enrichment analysis of miRNA targets in Hierarchical gene Co-expression signatures), to infer the perturbed miRNA regulatory networks by using the hierarchical co-expression signatures in large-scale cancer gene expression datasets. The method can infer onco-miR candidates and their target networks which are only linked to sub-clusters of the differentially expressed genes at fine scales of the co-expression hierarchy. On two real datasets of lung cancer and hepatocellular cancer, miRHiC uncovered several known onco-miRs and their target genes (such as miR-26, miR-29, miR-124, miR-125 and miR-200) and also identified many new candidates (such as miR-149, which is inferred in both types of cancers). Using hierarchical gene co-expression signatures, miRHiC can greatly increase the sensitivity for inferring the perturbed miRNA regulatory networks in cancer. All Perl scripts of miRHiC and the detailed documents are freely available on the web at http://bioinfo.au.tsinghua.edu.cn/member/jgu/miRHiC/.

## Introduction

MicroRNAs (miRNAs) are a class of small (∼22 nt) regulatory RNAs, which play important roles in many essential biological and physiological processes, such as embryo development, cancer progression and immune response. About 1,400 miRNAs have been identified in human and more than 30% known protein-coding genes are potentially regulated by evolutionary conserved miRNAs [1], [2]. The perturbations of some miRNAs, usually called as onco-microRNAs (onco-miRs, including both oncogenic and tumor suppressive miRNAs in this study), have been reported to be significantly associated with multiple stages of cancer. But till now, only a few of the hundreds of miRNAs are linked to the complex dys-regulated cellular processes in cancer. There is a great need for inferring the perturbed miRNA regulatory networks and their functions in cancer [3].

To infer the perturbed miRNA regulatory network, one popular strategy is to analyze miRNA target gene set enrichments in differentially expressed gene signatures. This includes many developed methods, such as gene set analysis by hyper-geometric test (HG-test, or Fisher's exact test); GSEA (gene set enrichment analysis) [4], [5]; FAME (functional assignment of miRNAs via enrichment) [6]; and miRBridge [7], which assume that the target gene set enrichments reflect the perturbations of their upstream miRNA regulation strengths. But due to the complexity of cancer transcriptome, these methods usually show low sensitivity of inferring onco-miR candidates (here, the “sensitivity” mainly means the number of inferred onco-miR candidates under a given statistical significance level).

Cancer is a multi-stage and mixed process, usually involving many hierarchically organized sub-processes regulated at multiple scales [8]. The miRNA regulations also show the property of multi-scale [9]: a few miRNAs, which help determine cell types or cellular states, suppress hundreds of target gene expressions to maintain cell type or cellular state specific expression profiles, such as miR-124 in brain and miR-1, miR-133 in muscle [10], [11], [12]; however, many other miRNAs may only regulate some specific processes by targeting a small group of closely-related genes. The former kind of candidate onco-miRs can be easily identified by analyzing the enrichment of their target genes in the whole set of the differentially expressed genes, but the latter ones are frequently missed by existing methods due to insufficient target gene enrichments in the differentially expressed genes or in the co-expression signatures using pre-defined similarity cutoffs.

In this study, we proposed a new strategy to infer the onco-miRs and their perturbed regulatory networks. This strategy takes consideration of the multi-scale and hierarchically organized regulatory structures in the differentially expressed genes using gene co-expression information, and fine-tunes the scales in the gene co-expression hierarchy to analyze the miRNA target gene set enrichment. Our method, named as miRHiC (enrichment analysis of miRNA targets in Hierarchical gene Co-expression signatures), can infer the perturbed miRNA regulatory networks in cancer by analyzing the enrichments of miRNA target gene sets in the hierarchical gene co-expression signatures. These gene signatures were established by hierarchical gene co-expression clustering, one common way to separate the mixed signals in gene expression profiles at different correlation levels. In miRHiC, the miRNA target gene set is not required to be enriched in the whole set of the differentially expressed genes but within any signature at the fine scale of the gene co-expression hierarchy. Besides the higher sensitivity for inferring the onco-miR candidates, another advantage of considering the gene co-expression information is to reduce the noises of inferring the corresponding perturbed target genes: the “scattered” differentially expressed genes with little expression pattern similarity to other genes, which are more likely to be “false” miRNA targets due to expression noises [13], are excluded during the analysis. On two large-scale cancer gene expression datasets, miRHiC successfully identified several known onco-miRs and also inferred many new candidates.

## Materials and Methods

### MiRNA target genes

miRNAs and their target genes (the miRNAs from the same family are merged as a single item) were extracted from TargetScan database (v6.2) [1], [2]. A gene was regarded as a target of one miRNA, if the gene contains at least one conserved predicted miRNA binding site in its 3′-UTR. And the summarized context score (a negative score measuring miRNA-target regulation strength or confidence, provided by TargetScan) was recorded for each miRNA-target pair. Then, we discretized the context scores into *K* levels: all miRNA-target pairs were sorted according to their context scores in decreasing order (the pairs ranked on the top have the lowest regulation strength) and the discretized score for the miRNA-target pair with rank *r* was defined as: *s* = 1+*b*[*rK*/*N*]. It means the first 1/*K* miRNA-target pairs have lowest score 1, while the last 1/*K* pairs have highest score 1+*b*(*K*-1). According to ref.[6], *K* is set as 5 and *b* as 3 in this study.

The control miRNA target gene sets were generated by bipartite graph based random permutation of the miRNA-target pairs with the same discretized scores but keeping the sizes of all target gene sets. This kind of stringent permutation procedure can generate the control miRNA target gene sets which preserve the statistical properties much better than the randomization without restriction [6].

### Cancer gene expression data

We test miRHiC on two large-scale cancer gene expression datasets downloaded from NCBI GEO database: 1) lung cancer (LUC) dataset, GSE19804 including 60 paired cancer and para-cancer samples; and 2) hepatocellular cancer (HCC) dataset, GSE22058 including 96 paired cancer and para-cancer samples. To avoid the noises in lowly expressed genes, we only kept the genes whose expression values rank at top 10,000 in at least 30% samples in each dataset. Then, the differentially expressed genes were identified with p-value <0.0001 using t-test (the p-values were multiple testing adjusted by BH correction). We identified 3,397 and 5,699 differentially expressed genes for LUC and HCC datasets, respectively.

### miRHiC: enrichment analysis of miRNA targets in Hierarchical gene Co-expression signatures

**Figure 1:**
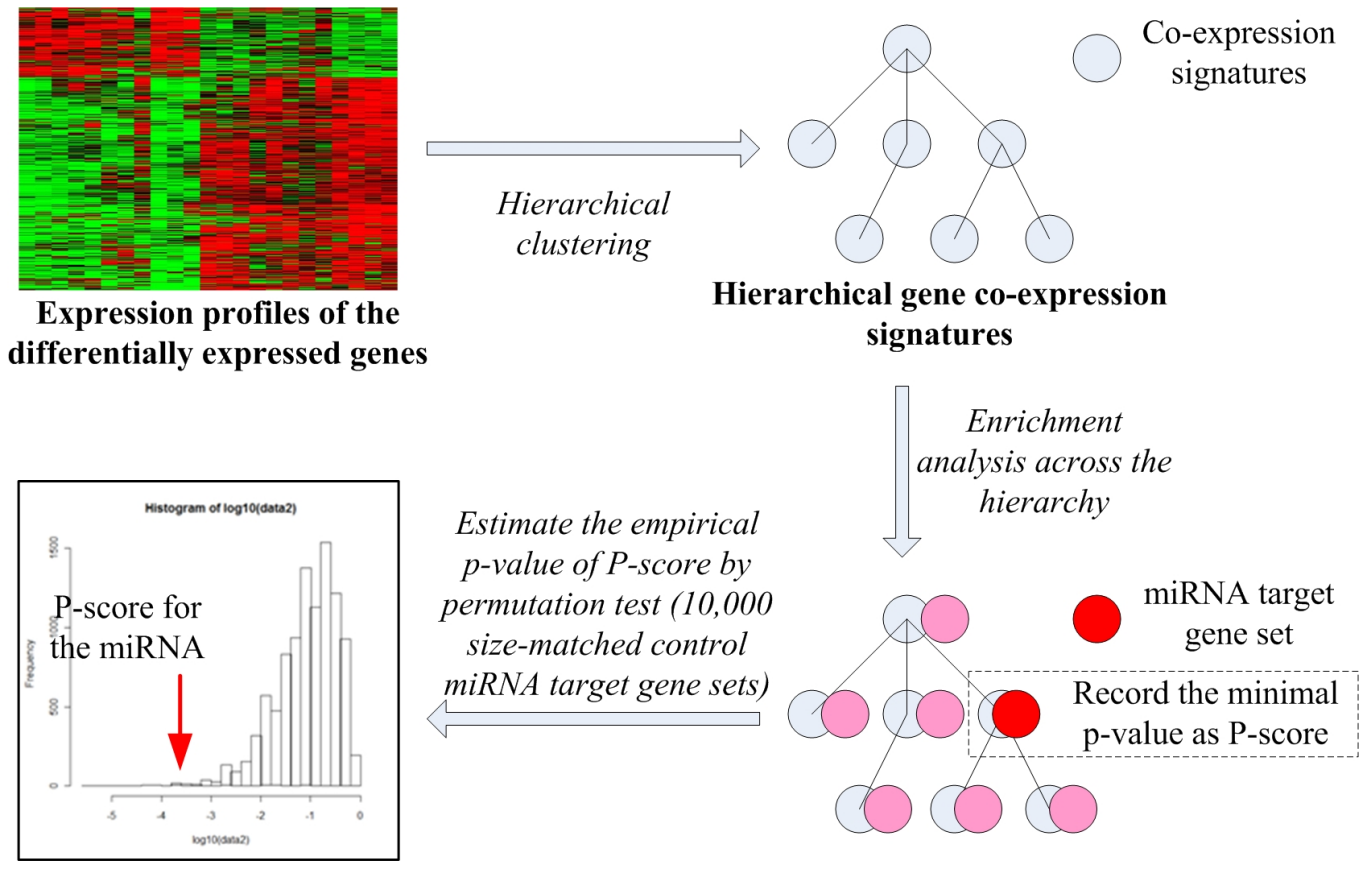
The flowchart of miRHiC. In the first step, the differentially expressed genes were clustered as hierarchical gene co-expression signatures; then, the most significant enrichment of the miRNA target gene set was found across the hierarchical signatures; and finally, a permutation test was used to estimate the empirical p-value of the enrichment.

miRHiC was proposed to infer the perturbed miRNA regulatory networks in cancer by incorporating the hierarchically organized co-expression information of the differentially expressed genes: firstly, the hierarchical gene co-expression signatures were established by clustering the differentially expressed genes based on pairwise gene co-expression correlations; then the miRNA target gene set enrichment was analyzed across the hierarchical co-expression signatures; and finally, a permutation test was used to estimate the statistical significance of the enrichment (Figure 1).

#### 1) Get the hierarchical gene co-expression signatures

Firstly, average linkage hierarchical clustering is implemented to cluster the differentially expressed genes based on their pairwise co-expression correlations. To reduce the noises caused by poorly correlated genes, the hierarchical clustering is stopped if the gene co-expression correlation is too low: we used the correlation with z-score 0.52 as the cutoff in this study (about p-value 0.3; z-score of any given correlation level is calculated using Fisher's transformation). This cutoff shows few influences on the results: for the LUC dataset, when the z-score cutoff changed from 0.3 to 0.9 by step 0.1, the hierarchical clustering was stopped at almost the same place. Then, we extracted the gene co-expression signatures (stable gene co-expression clusters) at different correlation scales by traversing the co-expression hierarchy from leaf to root (the correlation is decreasing and the size of signatures is increasing when traversing the hierarchy from leaf to root). The details of the signature extraction algorithm are given in user manual via miRHiC website.

#### 2) Analyze the miRNA target gene set enrichments in the hierarchical gene co-expression signatures

For the *j*-th gene co-expression signature in the hierarchy, we can find the overlapped genes between the signature (denoted as *S_j_*) and the *i*-th miRNA target gene set (denoted as *T_i_*), and then calculate the raw enrichment score by summing the discretized TargetScan scores (see the details of the score discretization in the above section) of the overlapped genes for *i*-th miRNA:
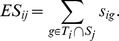



The p-value *p_ij_* for this enrichment was estimated by examining the enrichment scores *ES_ij_*(*r*) of 10,000 size-matched random control miRNA target gene sets:



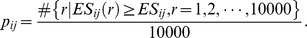



After getting the enrichments in all hierarchical gene co-expression signatures (*j* = 1, 2, …), the *P*-score *P_i_* for the *i*-th miRNA was calculated as the p-value of the most significant enrichment:
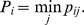



The *P*-score was used to measure the miRNA target gene enrichment across the whole gene co-expression hierarchy.

#### 3) Calculate the statistical significance of the *P*-score based enrichments

The *P*-score is the minimal of a set of p-values, so it is not uniformly distributed along 0∼1 (biased to 0). It cannot be directly used to measure the statistical significance of enrichment. Again, we used permutation test to estimate the statistical significance of the P-score: the P-scores *P_i_*(*r*) of 10,000 size-matched control miRNA target gene sets were calculated according to above steps; and the empirical p-value *p_i_* for the P-score *P_i_* was calculated as:
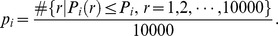



The empirical p-value *p_i_* was used to measure the statistical significance of miRNA target gene set enrichment across the whole hierarchical gene co-expression signatures. To correct the multiple test, fdrtool was used to calculate the *q*-values according to the empirical p-values [14].

### Comparisons with other methods

miRHiC was compared with Gene Set Enrichment Analysis (GSEA) and gene set analysis by hyper-geometric test (HG-test). GSEA is a widely used method for inferring the perturbed gene sets by taking the continuous values and the rank information of differential gene expressions [5]. When comparing miRHiC with GSEA, the fold changes of gene expressions between cancer and normal samples were used in GSEA and the same miRNA target gene set permutation method was used to calculate the empirical p-values.

GSEA and HG-test use different computational models to measure gene set enrichments with miRHiC. For directly testing the advantage of using the hierarchical gene co-expression information, we used the differentially expressed genes as the only signature and ran miRHiC on it. For clear presentation, we named this approach as miRDeG (enrichment analysis of miRNA targets in Differentially Expressed Genes).

Except hierarchical clustering, *k*-means clustering is another commonly used algorithm to generate gene co-expression signatures. The algorithm can partition all differentially expressed genes into *k* non-overlapped clusters. Unlike hierarchical clustering, *k*-means is hard to exclude the poorly correlated genes by setting any threshold. In the comparison, we used *k*-means (*k* is set to 5 or 10) to get the gene co-expression signatures. Then we run the same procedure to analyze the miRNA target enrichments in the generated signatures with different *k*. We named this approach as miRKM (miRKM5 and miRKM10) in the below section.

## Results

### Estimating the empirical p-values with no bias by miRHiC

**Figure 2:**
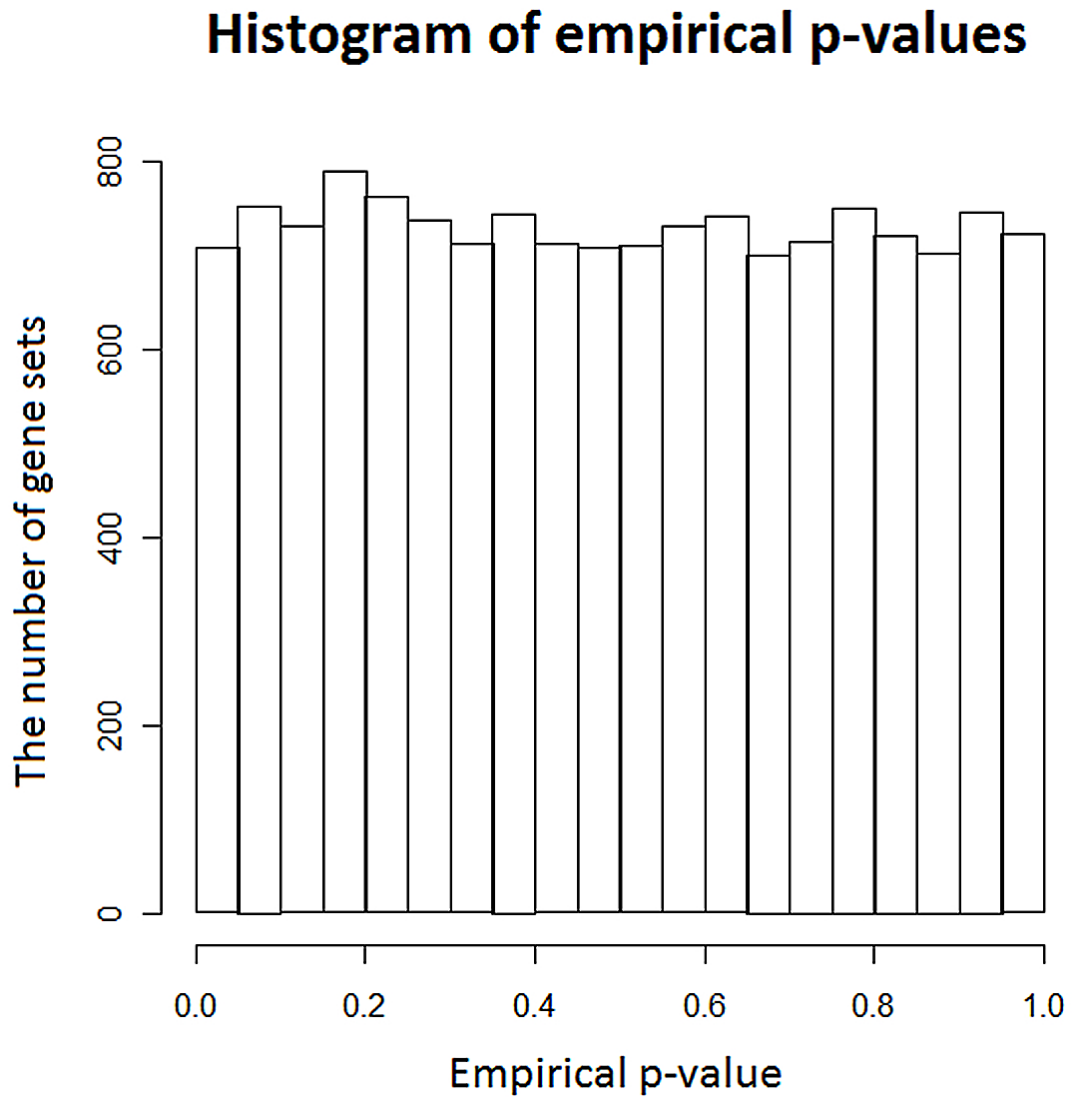
The histogram of the empirical p-values of the control miRNA target gene sets.

To demonstrate that miRHiC did not have the problem of over-estimating the statistical significances, we generated 100 size-matched control target gene sets for each miRNA, and then calculated distributions of the empirical p-values for their enrichments in the hierarchical gene co-expression signatures using miRHiC. If miRHiC has no bias for estimating the empirical p-values, the p-values of these control miRNA target gene sets should be uniformly distributed between 0∼1. As expected, results showed that the empirical p-values are uniformly distributed (Figure 2). Another possible bias affecting empirical p-value is caused by different sizes of miRNA target gene sets: some miRNAs have more than 1,000 target genes while some only have less than 50 target genes. We calculated the Spearman's rank correlation between the sizes and the corresponding empirical p-values of the gene sets. The correlation is −0.015 (p-value of this correlation >0.05), which suggested that the empirical p-values are not affected by the sizes of gene sets. Based on these analyses, we can conclude that miRHiC has no bias for estimating the empirical p-values.

### Inferring the perturbed miRNA regulatory networks in cancer

miRHiC can infer onco-miRs and their perturbed target regulatory networks by analyzing the miRNA target gene set enrichment in hierarchical gene co-expression signatures in cancer. On the two large-scale gene expression datasets of lung cancer (LUC) and hepatocellular cancer (HCC), miRHiC inferred 9 and 8 perturbed miRNAs or onco-miRs, respectively, with q-value <0.1. Under the same q-value cutoff, the three compared methods, GSEA, HG-test and miRDeG did not infer any candidate. Although miRKM inferred some candidates (for LUC dataset, miRKM5/10 inferred 3/4 candidates; and for HCC dataset, miRKM5/10 inferred 6/3 candidates), these numbers are still less than miRHiC and most of the miRKM inferences are covered by miRHiC. The details results are provided in Table S1. Among all 17 inferences from miRHiC, 9 are supported by direct functional evidences in literatures (LUC: miR-26, miR-29, miR-125, miR-130, miR-145 and miR-200; HCC: miR-21, miR-124 and miR-125). These results indicate that miRHiC can greatly improve the sensitivity of onco-miR inferences (Table 1). Considering the heterogeneity of cancer transcriptome, bootstrapping resampling was implemented to check the stability of the inferences. For LUC, 6 out of 9 candidates can be repeatedly inferred in more than 50% resampling experiments (miR-125, miR-149, miR-340 and miR-200 are stably inferred in more than 80% experiments). For HCC, 5 out of 8 candidates can be repeated inferred (miR-125 and miR-149 are stably inferred in more than 60% experiments).

**Table 1:** The onco-miRs inferred by miRHiC with q-value <0.1.

Cancer	miRNA	q-value a	References
LUC	miR-125	0 (BS b)	[29], [30], [31]
	miR-130	0 (BS)	[32]
	miR-340	0 (BS)	
	miR-874	0	
	miR-26	0.021 (BS)	[33], [34]
	miR-149	0.022 (BS)	
	miR-29	0.028	[35], [36], [37]
	miR-200	0.029 (BS)	[16], [38], [39], [40]
	miR-145	0.064	[41], [42], [43], [44], [45]
HCC	miR-125	0 (BS)	[46], [47], [48], [49]
	miR-149	0 (BS)	
	miR-370	0	
	miR-144	0.025	
	miR-339	0.026	
	miR-378	0.049	
	miR-21	0.058	[50], [51], [52], [53], [54], [55]
	miR-124	0.093	[56], [57]

a The q-value is due to empirical p-value <0.0001.

b (BS) labels mean that the miRNAs are inferred in more than 50% bootstrapping experiments with q-value <0.1.

**Figure 3:**
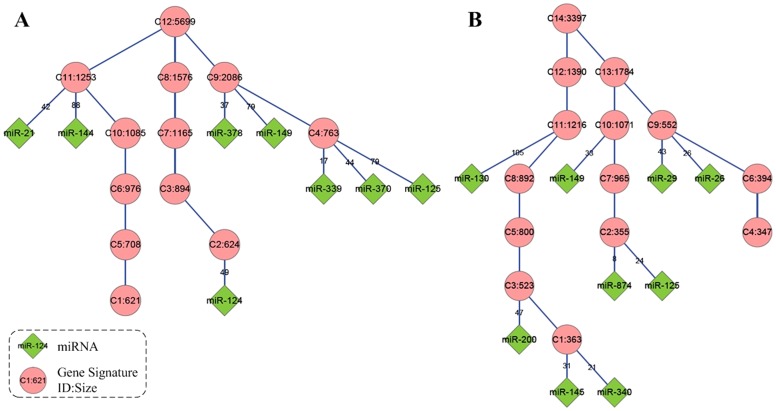
The perturbed miRNA regulatory networks in the two types of cancers inferred by miRHiC. A) is for lung cancer and B) for hepatocellular cancer. The circle nodes represent the gene co-expression signatures (ClusterID:Size). The diamond nodes represent the inferred onco-miRs. The numbers on the edges represent the sizes of the miRNA target genes overlapped with the corresponding gene co-expression signatures.

By looking at the targeted signatures of the inferred onco-miRs, we found that they have different levels of gene co-expressions in the hierarchies (Figure 3). The functions associated with these signatures (the enriched GO terms of the signatures were annotated by DAVID web tool [15]) are significantly related to different hallmarks of cancer, including cell cycle, oxidation reduction, immune response, DNA repair, cell adhesion and vasculature development (Table 2). These results indicate that many miRNAs are linked to cancer through different sub regulatory programs. For example, miR-200 is known as an important regulator of angiogenesis (a child term of “vasculature development”). There are several experimental validated target genes for angiogenesis, including ZEB1 and KDR [16], [17], [18], existing in the inferred perturbed miR-200 regulatory networks in LUC dataset. MiR-200 may regulate the angiogenic switch in lung cancer through these target genes. In hepatocellular cancer, miR-21 was predicted to regulate “immune response” by targeting CD69, STAT3, CCL20 and SMAD7, in which STAT3 and SMAD7 are important signaling molecules for immune response.

**Table 2:** The selected target genes and the related signature functions of the inferred onco-miR perturbed target networks.

Type	miRNA	Selected Targets a	Signatures and Functions
LUC	miR-125	—	C2 (355 genes): ncRNA processing (2.28e-3) b, oxidation reduction (9.09e-2)
	miR-874	—	
	miR-130	SERINC3, GJA1, PPARG, RTN1, BTG1, TGFBR2, TSC1, CD69, S1PR1, TNFRSF1B, ZEB2	C11 (1216 genes): vasculature development (3.77e-6), cell adhesion (6.80e-6), lung development (1.13e-5)
	miR-340	TNS1	C1 (363 genes): cell adhesion (3.82e-7), vasculature development (4.35 e-7)
	miR-145	LYVE1, NEDD9, AKAP12, CAV2	
	miR-26	KMT6, SULF1, HMGA1	C9 (552 genes): cell cycle (1.99e-6), DNA repair (1.63e-15)
	miR-29	COL1A1, MEST, MYBL2, PDGFC, GEMIN2, GPI	
	miR-149	MMP15, ENC1	C10 (1071 genes): ncRNA metabolic process (2.48e-4), oxidation reduction (1.50e-2)
	miR-200	ZEB1, KDR, TBX5, CHRDL1	C3 (523 genes): cell adhesion (1.81e-9), vasculature development (1.73e-8)
HCC	miR-125	ERBB3, NEU1, RAF1, MAP2K7	C4 (763 genes): chromatin modification (6.66e-5), regulation of transcription (2.27e-3)
	miR-370	SMO, HDAC4	
	miR-339	NF2, THRA	
	miR-149	CLTC	C9 (2086 genes): cell cycle (2.22e-12), DNA repair (9.72e-4)
	miR-378	MAPK1	
	miR-144	ETS1, CXCL12, GATA3, PIM1, PODXL, RARB	C11 (1253 genes): immune response (6.53e-49), cell adhesion (3.87e-21), cell activation (1.60e-17)
	miR-21	SMAD7, CCL20, ARHGAP24, CD69, SPRY2, RHOB, STAT3	
	miR-124	CPT1A, CYB5A, RAPH1, SORD, ALDH9A1, AR, HADHA	C2 (624 genes): oxidation reduction (7.67–42)

a The selected targets with high TargetScan scores and literature evidences (reported in at least 5 PubMed abstracts with key words “liver cancer” or “lung cancer”).

b These functional terms (and the corresponding FDRs) are the selected top enriched GO terms of the signature annotated by DAVID web tool.

### Perturbed miR-149 sub-networks shared by the two types of cancers

**Figure 4:**
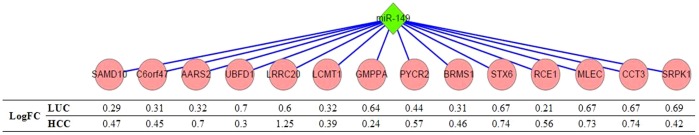
The perturbed miR-149 sub-networks shared by LUC and HCC. The average log-transformed fold changes of the shared target genes are also shown in the below table.

The onco-miRs inferred in multiple cancers may play more important roles in cancer initiation and development. Two miRNAs, miR-125 and miR-149 were inferred by miRHiC in both types of cancers. For the inferred perturbed miR-125 regulatory networks, there are only three common targets (CDK16, TOMM40 and KIAA1522), which suggests that miR-125 may regulate different pathways in the two types of cancers. While for miR-149, its perturbed regulatory networks show significant target overlapping with a shared sub-network including 14 common targets. And the 14 targets are consistently over-expressed in cancer tissues (Figure 4).

MiR-149 is a mammal conserved miRNA. A few studies show that miR-149 genetic polymorphisms are associated with the risk of cancer [19], [20]. Its expression is epigenetic silenced by DNA hyper-methylation in colorectal cancer [21]. But the miR-149 regulatory networks are still poorly understood in cancer. The inferred perturbed networks provide important insight of miR-149 regulations: most of the high confidence targets (with high TargetScan scores) in the shared sub-network are related to some essential biological processes, such as SRPK1 (serine/arginine-rich splicing factor kinase 1) and CCT3 (chaperonin containing TCP1, subunit 3). SRPK1 encodes a serine/arginine protein kinase specific for the SR (serine/arginine-rich domain) family of splicing factors. SRPK1 is upregulated in lung cancer and many other cancer types [22], [23]. CCT3 is a subunit of a molecular chaperone protein (chaperonin containing TCP1 complex) helping fold actin/tubulin and it can positively regulate cell cycle [24], [25]. CCT3 over-expression is also reported to be related to colorectal cancer [26] and liver cancer [27]. So, miR-149 may work as a cancer suppressor by targeting these oncogenes.

## Discussion

Analyzing miRNA target gene set enrichment in differentially expressed genes of large-scale gene expression profiles can greatly advance our understandings of the perturbed miRNA regulations. But due to the complexity of cancer transcriptome, it is challenging to infer the perturbed miRNA regulations by simply analyzing miRNA target gene set enrichment in the whole differentially expressed genes. In this study, we developed miRHiC to infer the perturbed miRNA regulatory networks in cancer by incorporating the hierarchical gene co-expression information into miRNA target gene set enrichment analysis. Results showed that miRHiC have much higher sensitivity for the inferences than the commonly used methods, such as HG-test, GSEA and miRDeG (FAME), of which all do not use the hierarchical gene co-expression information. Over 50% of the inferred onco-miRs have extensive literature supports and the gene co-expressions signatures targeted by these miRNAs are significantly related to multiple hallmarks of cancer. Recent studies also show that gene co-expressions can provide important information to identify the “real” target genes of miRNAs in the corresponding biological process [13], [28], which suggest that the target genes overlapped with the enriched co-expression signatures are more likely the real targets in cancer. Although miRHiC improved the sensitivity for inferring the onco-miRs and their perturbed target networks, a few known onco-miRs, such as miR-126 in lung cancer and miR-122 in hepatocellular carcinoma, were missed. These missed cases suggest that other computational models need to be developed for identifying the onco-miRs whose regulatory networks cannot be explained by the target gene enrichments in differential gene expression signatures.

The lengths of 3′-UTRs are strongly correlated with the number of targeted miRNAs and the context scores. The enriched signatures may significantly be biased to the ones with longer 3′-UTRs. When using hyper-geometry test to analyze the enrichments of miRNA target gene sets, we found that the signatures targeted by the inferred miRNAs have much longer average lengths of 3′-UTRs. However, like FAME [6], miRHiC used the bi-partite graph based permutation method, which can largely reduce this bias: the average lengths of 3′-UTRs of the genes in the signatures targeted by the inferred onco-miRs are 1314 nt and 1449 nt for the LUC and HCC datasets, respectively, not longer than these lengths of the differentially expressed genes (1424 nt and 1470 nt, respectively).

miRHiC provides a general strategy to analyze miRNA regulations using hierarchical signatures. Different hierarchical clustering methods can be used to get the hierarchical gene co-expression signatures. Besides the gene co-expression, the functional and regulatory interactions between genes (such as protein-protein interactions, transcriptional regulations and literature co-occurrences) can further be integrated to establish the hierarchical gene signatures. We will continuously test miRHiC strategy using different kinds of implementations.

To get better control sets of the miRNA target gene sets, miRHiC used the bi-partite graph based permutation. But this permutation method is time-consuming. Also, the computational burden is high for calculating the empirical p-values in a nested manner across the hierarchical gene co-expression signatures. We plan to develop faster algorithm to reduce the redundant calculations for estimating the p-values in future.

## Supporting Information

Table S1 The detailed results of miRHiC, GSEA, HG-test, miRDeG and miRKM.
(XLSX)
